# Real-life comparison of mortality in patients with SARS-CoV-2 infection at risk for clinical progression treated with molnupiravir or nirmatrelvir plus ritonavir during the Omicron era in Italy: a nationwide, cohort study

**DOI:** 10.1016/j.lanepe.2023.100684

**Published:** 2023-07-14

**Authors:** Carlo Torti, Pier Paolo Olimpieri, Paolo Bonfanti, Carlo Tascini, Simone Celant, Danilo Tacconi, Emanuele Nicastri, Evelina Tacconelli, Bruno Cacopardo, Alessandro Perrella, Giovanni Battista Buccoliero, Giustino Parruti, Matteo Bassetti, Carlo Biagetti, Andrea Giacometti, Elke Maria Erne, Maria Frontuto, Massimiliano Lanzafame, Valentina Summa, Alessandra Spagnoli, Annarita Vestri, Giovanni Di Perri, Pierluigi Russo, Giorgio Palù

**Affiliations:** a Department of Medical and Surgical Sciences, “Magna Graecia” University, Catanzaro, Italy; b Italian Medicines Agency, Via del Tritone 181, 00187 Rome, Italy; c Department of Public Health and Infectious Diseases, Sapienza University of Rome, Rome, Italy; d Fondazione IRCCS San Gerardo dei Tintori, University of Milano-Bicocca, Monza, Italy; e Department of Medicine (DAME), Infectious Diseases Clinic, Udine University Hospital, Udine, Italy; f Department of Specialised and Internal Medicine, Infectious Diseases Unit, San Donato Hospital, Arezzo, Italy; g National Institute for Infectious Disease Lazzaron Spallanzani, IRCCS, Via Portuense 292, 00149, Rome, Italy; h Infectious Diseases, Department of Diagnostic and Public Health, University of Verona, 37129 Verona, Italy; i Department of Internal and Experimental Medicine, University of Catania School of Medicine, Catania, Italy; j Division Emerging Infectious Disease and High Contagiousness, D. Cotugno Hospital, 80131 Naples, Italy; k Infectious Diseases Unit, San Giuseppe Moscati Hospital, Azienda Sanitaria Locale Taranto, 74121 Taranto, Italy; l Department of Medicine, Infectious Disease Unit, Pescara General Hospital, Pescara, Italy; m Department of Health Sciences (DISSAL), University of Genoa, Genoa, Italy; n Infectious Diseases Unit, Policlinico San Martino Hospital—IRCCS, Genoa, Italy; o Unit of Infectious disease Infermi Hospital, AUSL Romagna, Rimini, Italy; p Azienda Ospedaliera Universitaria, Ospedali Riuniti di Ancona, Ancona, Italy; q Department of Infectious Disease, Azienda Sanitaria dell’Alto Adige, Central Hospital of Bolzano, Italy; r Infectious Diseases Unit, A.O.R. San Carlo, Potenza, Italy; s Department of Medical, Infectious Diseases Unit, Santa Chiara Hospital, Trento, Italy; t Department of Medical Sciences at the Unit of Infectious Diseases, University of Torino, Amedeo di Savoia Hospital, Torino, Italy

**Keywords:** COVID-19, SARS-CoV-2, Nirmatrelvir/ritonavir, Molnupiravir, Real-world, Effectiveness

## Abstract

**Background:**

Comparative data on mortality in COVID-19 patients treated with molnupiravir or with nirmatrelvir plus ritonavir are inconclusive. We therefore compared all-cause mortality in community-dwelling COVID-19 patients treated with these drugs during the Omicron era.

**Methods:**

Data collected in the nationwide, population-based, cohort of patients registered in the database of the Italian Medicines Agency (AIFA) were used. To increase completeness of the recorded deaths and date correctness, a cross-check with the National Death Registry provided by the Ministry of the Interior was performed. We included in this study all patients infected by SARS-CoV-2 treated within 5 days after the test date and symptom onset between February 8 and April 30, 2022. All-cause mortalities by day 28 were compared between the two treatment groups after balancing for baseline characteristics using weights obtained from a gradient boosting machine algorithm.

**Findings:**

In the considered timeframe, 17,977 patients treated with molnupiravir and 11,576 patients with nirmatrelvir plus ritonavir were included in the analysis. Most patients (25,617/29,553 = 86.7%) received a full vaccine course including the booster dose. A higher crude incidence rate of all-cause mortality was found among molnupiravir users (51.83 per 100,000 person-days), compared to nirmatrelvir plus ritonavir users (22.29 per 100,000 person-days). However, molnupiravir-treated patients were older than those treated with nirmatrelvir plus ritonavir and differences between the two populations were found as far as types of co-morbidities were concerned. For this reason, we compared the weight-adjusted cumulative incidences using the Aalen estimator and found that the adjusted cumulative incidence rates were 1.23% (95% CI 1.07%–1.38%) for molnupiravir-treated and 0.78% (95% CI 0.58%–0.98%) for nirmatrelvir plus ritonavir-treated patients (adjusted log rank p = 0.0002). Moreover, the weight-adjusted mixed-effect Cox model including Italian regions and NHS centers as random effects and treatment as the only covariate confirmed a significant reduced risk of death in patients treated with nirmatrelvir plus ritonavir. Lastly, a significant reduction in the risk of death associated with nirmatrelvir plus ritonavir was confirmed in patient subgroups, such as in females, fully vaccinated patients, those treated within day 2 since symptom onset and patients without (haemato)-oncological diseases.

**Interpretation:**

Early initiation of nirmatrelvir plus ritonavir was associated for the first time with a significantly reduced risk of all-cause mortality by day 28 compared to molnupiravir, both in the overall population and in patient subgroups, including those fully vaccinated with the booster dose.

**Funding:**

This study did not receive funding.

**Research in context**

**Evidence before this study**

Emergency authorization of molnupiravir and nirmatrelvir plus ritonavir has been released for the treatment of non-hospitalised persons with symptomatic COVID-19 at high risk for progression to severe COVID-19 after the results of the MOVe-OUT and EPIC-HR trials became available. However, both trials enrolled unvaccinated patients with mild-to-moderate COVID-19 during a pandemic wave of the SARS-CoV-2 delta (B.1.617.2) variant, therefore their results cannot be transferred to current clinical practice. We searched Scopus, PubMed, and major databases of preprint publications for real-life studies published before February 28, 2023, using the search terms “SARS-CoV-2 OR COVID-19” AND “molnupiravir OR Lagevrio OR EIDD-2801” OR “nirmatrelvir OR Paxlovid OR PF-07321332”. We found only three major studies (conducted respectively in the U.S., in Greece and in Hong Kong) aimed at providing an adjusted comparison between the oral antivirals molnupiravir and nirmatrelvir plus ritonavir, none reaching statistically significant results in demonstrating a real benefit of one drug over the other as far as patient survival was concerned. Therefore, a valid assessment on a hard end-point such as death in those patients treated with the oral antivirals nirmatrelvir plus ritonavir and molnupiravir provided inconclusive results to inform their clinical use in patients with COVID-19, considering patients’ vaccination status and the circulating variant of concern.

**Added value of this study**

Our prospective, nationwide, cohort study adds significance to the literature on real-world effectiveness of nirmatrelvir plus ritonavir relative to molnupiravir because among the adequately powered studies, it was the first to demonstrate an added benefit of nirmatrelvir plus ritonavir on patient survival in a population of mainly vaccinated patients in the Omicron era. To improve validity of the study with complete recording of the death events and their dates, the Italian Medicines Agency registry was linked to the national register office for the resident population, which is a central database upheld by the Ministry of the Interior of Italy. The crude incidence rate of all-cause mortality at day 28 was 40.23 per 100,000 person-days among the overall population. A higher crude incidence rate of all-cause mortality was found among molnupiravir users (51.83 per 100,000 person-days), compared to nirmatrervir/ritonavir users (22.29 per 100,000 person-days). Comparing the weight-adjusted cumulative incidences using the Aalen estimator, we found that by day 28 rates were 1.23% (95% CI 1.07%–1.38%) for molnupiravir and 0.78% (95% CI 0.58%–0.98%) for nirmatrelvir plus ritonavir (p = 0.0002). Moreover, to account for possible heterogenicity, a weight-adjusted mixed-effect Cox model was performed, including Italian regions and centers as mixed-effects and treatment as the only covariate. Using this method, we found that nirmatrelvir plus ritonavir was associated to a significant reduction in the risk of death by day 28 compared to molnupiravir (HR 0.68 [95% CI 0.56–0.83]). Lastly, reduced risk of mortality associated with the use of nirmatrelvir plus ritonavir compared to molnupiravir was consistently observed in the overall population and in most of the patient subgroups using a stringent method (Holm-Bonferroni correction), reinforcing the additional benefit of early use of nirmatrelvir plus ritonavir over molnupiravir in reducing all-cause mortality.

**Implications of all the available evidence**

The present study provides strong support to nirmatrelvir plus ritonavir rather than molnupiravir as a preferred option for early treatment of SARS-CoV-2 infected patients at risk of clinical progression notwithstanding receipt of a full vaccine course in the Omicron era. More strategic studies are needed to elucidate the added benefit of mortality from the use of nirmatrelvir plus ritonavir in patient subgroups and in other patient populations and healthcare settings to further validate our results.

## Introduction

Since January 2020, almost 7 million people have died due to the coronavirus disease-2019 (COVID-19).^1^ COVID-19 can rapidly progress and several patients require hospitalisation and intensive care, while most people remain asymptomatic. Severe disease due to COVID-19 is associated with older age, obesity, and several chronic co-morbidities, including cardiovascular, kidney, and pulmonary diseases.^2^^,^^3^

Emergency authorisation of several therapies had been provided for the treatment of non-hospitalised persons with symptomatic COVID-19 at high risk for progression to severe COVID-19. The only two antiviral drugs available for oral administration - nirmatrelvir co-formulated with the boosting agent ritonavir (nirmatrelvir plus ritonavir) as well as molnupiravir—had received emergency authorisation^4^ since a significant reduction in COVID-19-related hospitalisation or death was demonstrated in early randomized controlled trials (RCTs) with these drugs compared to placebo.^5^^,^^6^

A systematic review and network meta-analysis^7^ of three randomized controlled trials^5^^,^^6^^,^^8^ indicated that, compared to placebo, nirmatrelvir plus ritonavir was associated with the lowest risk of hospitalisation or death (odds ratio, OR 0.12 [95% CI 0.06–0.24]), followed by remdesivir (OR 0.13 [95% CI 0.03–0.57]) and subsequently molnupiravir (OR 0.67 [95% CI 0.46–0.99]).

Besides remdesivir, which is currently available only for intravenous administration, estimates of the effectiveness of oral antivirals in real word cohorts are unreliable in most studies due to the small number of patients enrolled.^9^, ^10^, ^11^, ^12^, ^13^, ^14^, ^15^, ^16^, ^17^, ^18^ However, four studies^19^, ^20^, ^21^, ^22^ were adequately powered to provide reliable results. The study conducted in Hong Kong by Wong et al.^19^ enrolled in the first semester of 2022 mostly older people, non-hospitalized, with multiple pre-existing co-morbidities. In this study, early initiation of both antivirals was associated with reduced risk of mortality and in-hospital disease progression. Although a direct comparison between the two groups was not attempted after controlling for potential confounders, patients treated with nirmatrelvir plus ritonavir showed a lower risk of hospitalisation in contrast to those treated with molnupiravir. However, in the same study, only a small number of patients were fully vaccinated with the primary series and individuals in residential care homes for elderly were excluded. Xie et al.^20^ conducted an analysis on the US Department of Veterans Affairs database and found that molnupiravir was associated with a reduction in hospital admission or death at 30 days compared to no treatment. Lastly, two large cohort studies^21^^,^^22^ were conducted in the database of the largest healthcare provider in Israel through a propensity score matched analysis of patients who were treated with either nirmatrelvir plus ritonavir^21^ or molnupiravir^22^ in comparison to patients who did not receive these drugs. In these studies, both nirmatrelvir plus ritonavir^21^ and molnupiravir^22^ appeared to be effective in reducing the risk of severe COVID-19 and mortality, although the former drug appeared to be more effective in older patients, immunosuppressed patients, and patients with underlying neurological or cardiovascular disease, while the latter was associated with a significant decrease in the risk of the composite outcome in older patients, in females, and in patients with inadequate COVID-19 vaccination.

Only few cohort studies attempted to compare the clinical outcomes of patients after nirmatrelvir plus ritonavir or molnupiravir in the real life using appropriate statistical methods to minimise the risk of bias, which may be particularly due to confounding by indication. A study conducted among U.S. Veterans whom received COVID-19 antivirals within 10 days after the test-positive date demonstrated that there was a not statistically significant reduction in absolute risk of 30-day rate of death among patients treated with nirmatrelvir plus ritonavir compared to molnupiravir-treated participants (−7.89 events per 1000 patients), with a 0.14 relative risk reduction [95% CI 0.02–1.16]).^23^ A nationwide cohort study conducted in Greece^24^ showed that patients treated with nirmatrelvir plus ritonavir had a lower relative risk for hospital admission compared to those treated with molnupiravir, however statistical significance was not met for the odds ratio of death (OR 0.69 [95% CI 0.46–1.06], p = 0.09). Lastly, Wai et al.^25^ examined two outpatient and inpatient cohorts in Hong Kong. Both nirmatrelvir plus ritonavir and molnupiravir were associated with significantly lower risks of death in the inpatient cohort using inverse probability of treatment weighting to adjust patient characteristics (nirmatrelvir plus ritonavir: HR 0.10 [95% CI 0.05–0.21], p < 0.0001; molnupiravir: HR 0.31 [95% CI 0.24–0.40], p < 0.0001). In the outpatient cohort, a survival analysis was not performed due to the small number of observed deaths and the high risk of providing inconclusive results.

The present study was designed to perform a comparison for the main outcome of all-cause mortality between patients treated with molnupiravir or nirmatrelvir plus ritonavir within 5 days after the test-positive date and symptom onset in a nationwide, population-based, cohort of COVID-19 outpatients registered in the database of the Italian Medicines Agency (AIFA).

## Methods

### Study design and data sources

This is a nationwide prospective cohort study including all non-hospitalized patients aged 18 years or older with confirmed SARS-CoV-2 infection, treated in Italy with molnupiravir or nirmatrelvir plus ritonavir oral antiviral agents between February 8, 2022 and April 30, 2022. During this timeframe, Italy was mainly characterized by the BA.1, BA.1.1, and BA.2 Omicron variants/subvariants, with the first two (BA.1, BA.1.1) that were deemed to be responsible for almost 90% of infections in February 2022 and the third (BA.2) for >70% cases in April 2022.^26^

Data were collected from the AIFA web platform of Monitoring Registries (wMRs), an administrative database whose main purpose is to provide authorization for use and monitoring the appropriateness of drug prescription in Italy. In fact, wMRs have been already widely used in Italy to monitor drug utilization and effectiveness in the clinical practice.^27^, ^28^, ^29^, ^30^ Data were collected to evaluate eligibility for treatment in patients and collect information on treatment effectiveness. In this perspective, clinicians were required to fill in the following information: date of symptom onset**,** COVID-19 severity and type of symptoms, risk factors associated with evolution to severe COVID-19 including co-morbidities, pregnancy status, need of supplemental oxygen therapy, vaccination status and date of last administration, oxygen saturation, hepatic and renal function, demographic information (age, sex), dates of drug prescription and dispensation. Given the administrative and mandatory nature of the wMRs, no missing data were present among the collected baseline characteristics.

After 30 days of treatment initiation, clinicians were requested to fill in an end-of-treatment report, which includes information about the treatment outcomes (recovery or death, death date and drug tolerability). Nevertheless, given the high patient load on the National Health Service (NHS) centers in the considered timeframe, many end-of-treatment forms were not reported, and, they were missing at the time of data collection. Nevertheless, for the primary outcome, the death dates of patients included in the registry were obtained from the national register office for the resident population (ANPR, available at https://www.anagrafenazionale.interno.it/), which is a central database upheld by the Ministry of the Interior of Italy (decree 82/2005, art. 62). By Italian Laws (DPR 3 November 1990, n. 396), death certificates must be uploaded in the ANPR within 10 days from the event and data extraction was performed on December 14, 2022, therefore patient status (alive or death) at day 28 and date of death were available for all treated patients included in the study.

It is important to note that from January 2022 to May 2022, patient inclusion in the wMRs was mandatory for clinicians to prescribe molnupiravir or nirmatrelvir plus ritonavir. Two important implications followed this requirement: *i*) patients were prospectively followed for one month since drug dispensation and, *ii*) the included patients represented a census of the SARS-CoV-2 infected population treated between February 2022 and April 2022 in Italy with oral antivirals.

In Italy, the following criteria are mandatory for a patient to be eligible for treatment with oral antivirals: confirmed SARS-CoV-2 infection; mild to moderate COVID-19; non-hospitalized or hospitalized for reasons unrelated to SARS-CoV-2 infection; early stage of disease (within 5 days of symptoms onset unless otherwise clinically indicated); no severe hepatic or renal impairments (for nirmatrelvir plus ritonavir only); at least one risk factor associated with evolution to severe COVID-19 (unless otherwise clinically indicated). Risk factors associated with evolution to severe COVID-19 included: *i*) oncological or onco-haematological diseases, henceforth indicated as (haemato)-oncological diseases; *ii*) chronic kidney diseases; *iii*) severe pulmonary diseases; *iv*) primary or acquired immunodeficiencies; *v*) obesity (BMI >30 kg/m^2^); *vi*) cardio- and cerebro-vascular diseases; *vii*) decompensated diabetes mellitus (HbA1c > 9.0% or > 75 mmol/L).

Moreover, we excluded from our study patients: *i*) with severe hepatic or renal impairment (for molnupiravir only); *ii*) treated with other antiviral or monoclonal antibody therapies within 28 days before or after oral antiviral treatment; *iii*) with no risk factors other than age; *iv*) with more than 5 days between symptoms onset and treatment start or with more than 5 days between the date of the positive test and treatment start.

### Procedures

Oral antiviral posology was defined according to the recommendations reported in the summary of product characteristics (nirmatrelvir plus ritonavir) and the European Medicines Agency (EMA) conditions of use for unauthorized product (molnupiravir): 300 mg nirmatrelvir + 100 mg ritonavir twice daily for 5 days or 150 mg nirmatrelvir + 100 mg ritonavir twice daily for 5 days in case of moderate renal impairment (eGFR from ≥30 to < 60 mL/min) or 800 mg molnupiravir twice daily for 5 days.

The decision to prescribe molnupiravir rather than nirmatrelvir plus ritonavir was taken by the single specialist according to international guidelines available online (e.g., https://www.idsociety.org/practice-guideline/covid-19-guideline-treatment-and-management), the indications set by the Italian Ministry of Health (available at: https://portale.fnomceo.it/wp-content/uploads/2022/02/Com-40_signed.pdf) and her/his best clinical judgement, considering for example the risk of drug–drug interactions (usually checked at: https://www.covid19-druginteractions.org/). However, in the study period, access to either drug was comparable since both molnupiravir and nirmatrelvir plus ritonavir were dispensed from authorised centres under the responsibility of prescribing physicians designated by the Regional Health Authorities.

COVID-19 cases were defined to be mild in presence of symptoms such as: fever, malaise, pharyngodynia, nasal congestion, headache, myalgias, diarrhea, anosmia, dysgeusia, in the absence of dyspnoea, dehydration, altered state of consciousness following the indications of the Italian Ministry of Health (available at: https://portale.fnomceo.it/wp-content/uploads/2022/02/Com-40_signed.pdf). No information regarding hospitalisation were available since receiving the study drugs while inpatient for reasons unrelated to COVID-19 was considered as an inclusion criterium together with being outpatients (*i.e.*, inpatient or outpatient status were not inputed as separate records in the database).

Molnupiravir and nirmatrelvir plus ritonavir all-cause mortality by day 28 was compared after balancing for baseline characteristics and regardless of patient adherence to treatment.

### Ethical statement

According to decree 196/2003 (“Italian Privacy Code”) and decree 101/2018 (“Harmonization Decree” harmonizing the Italian data protection laws with the provision of the General Data Protection Regulation 679/2016—GDPR), the processing of anonymized data does not require authorisation by patients if carried out in the performance of public interest or public powers based on a provision of law.

### Outcomes

All-cause mortality after 28 days from initial drug administration was the primary outcome collected in the overall population and compared between the two groups. First day of drug administration was labeled as day 0; all patients without events were then censored after day 28 (labeled as day 27). Tolerability profiles were assessed only when the end of treatment form was available for each single patient.

### Statistical analysis

Balancing between the two groups was performed using weights obtained from a gradient boosting machine algorithm (gbm).^31^ In brief, an Inverse Probability Treatment Weighting (IPTW) approach was adopted, estimating propensity scores from the tree-based gbm method and calculating the relative weights. This type of models has the advantage of automatically incorporating non-linearities and interactions among the covariates. The weights were then used to adjust Aalen estimates of the cumulative incidence of death between the two groups and to fit mixed effect Cox proportional hazard models both in the overall population and in the subgroups of interest, to obtain the hazard ratios and their 95% confidence intervals of the comparison nirmatrelvir plus ritonavir versus molnupiravir. Start of follow-up was the first day of drug administration and end of follow up was day 28 after treatment initiation. All deaths occurring during the follow-up were considered as events no matter the cause. Since we had a 100% capture of all deaths occurring during the study period (as well as in the next 6–7 months), no patients were censored up to the end of the follow-up period (non-informative type 1 censors).

Noteworthy, since significant heterogeneity in patient characteristics and outcomes may be present among Italian regions,^32^ the mixed effect was performed to account for the underlying variation between Italian regions and among the NHS centers.

More in detail, the gradient boosting model was built using the R statistical software gbm package (available at: https://CRAN.R-project.org/package=gbm) with the following parameters: Bernoulli distribution, 10,000 trees, 4 levels of variable interactions and a shrinkage parameter of 0.1. The variables used for estimating the propensity scores were: age class (54 years old or younger; from 55 years to 74 years old; 75 years old or older); sex; severity of the clinical syndrome (as per clinical judgement); vaccination status (none, partial and full when it included the booster dose), type of vaccine administered and days from last vaccine administration; non-COVID-19 related oxygen therapy (Yes or No); hepatic function (normal, Child Pugh A and Child Pugh B, labelled, respectively, as normal, mild and moderate impairment) and renal function (eGFR ≥90, eGFR ≥60 and < 90 mL/min and eGFR da ≥30 a <60 mL/min, labeled, respectively, as normal, mild or moderate impairment); presence/absence of the aforementioned risk factors (one binary variable for each recorded risk factor); days since symptom onset. We also defined and included in the gbm model a new variable labeled week-month reflecting the date (express as month and week) of the positive SARS-CoV-2 test performed, as a proxy for SARS-CoV-2 variant, which was not available.

The propensity scores and relative weights were obtained using the R twang package^32^; the same library was also adopted for model diagnostic and variable balancing check using the standardized effect size and maximum pairwise Kolmogorov–Smirnov statistic (see Supplementary Figures S1–S4). More specifically the standardized mean differences before and after adjusting with the obtained weights were compared between the two cohorts, in the overall population and in the subgroups of interest (see Supplementary Figures S5 and S6 and Supplementary Table S1). Weight-adjusted Aalen estimates were obtained using the svykm function of the R survey library (available at: https://CRAN.R-project.org/package=survey), which adopted the Aalen rather than the Kaplan–Meier estimator when standard errors have to be computed. Log rank test was corrected using Jun Xie and Chaofeng Liu method.^33^

Adjusted cumulative incidences were calculated multiplying each patient follow-up period (28 days in the case of survivors or the time to death for deceased patients) for its weight. Number of events were then divided for the sum of the overall adjusted person-days and reported as number of events per 100,000 person-days. Mixed effects Cox models (univariable, age-adjusted and including all selected variables at once) were adopted to investigate the association between specific baseline characteristics and mortality by day 28 using the coxme R library^34^ in the overall population.

Different univariable mixed effects Cox models, relating the effect of treatment on survival time, were performed^34^ for the overall population and the following subgroups: patients aged 55–74 years, patients aged 75 year or more, men, women, fully vaccinated, partially or non-vaccinated patients, patients started up to 2 days since symptoms onset, patients started from 3 to 5 days since symptoms onset, oncological or onco-hematological patients and non-oncological and non-onco-hematological patients. Mixed effects were included in the models after rejecting the null hypothesis of a likelihood-ratio test comparing models with or without mixed effects. Proportional hazard assumptions were checked by testing for independence between Schoenfeld residuals and time and by graphical inspection (see Supplementary Table S2 and Supplementary Figure S7). Moreover, since we performed several different Cox models, we also decided to account for multiple comparisons using the Holm-Bonferroni method.

All statistical analyses were performed using R, version 4.2.0 (R Core Team, 2014). Numerical variables were described using median with first and third quartile (q1-q3) values, categorical variables were described using frequencies. All significance tests were two-tailed, and a p-value of less than 0.05 was statistically significant.

### Role of the funding source

No fundings were received for the present study.

## Results

**Fig. 1:**
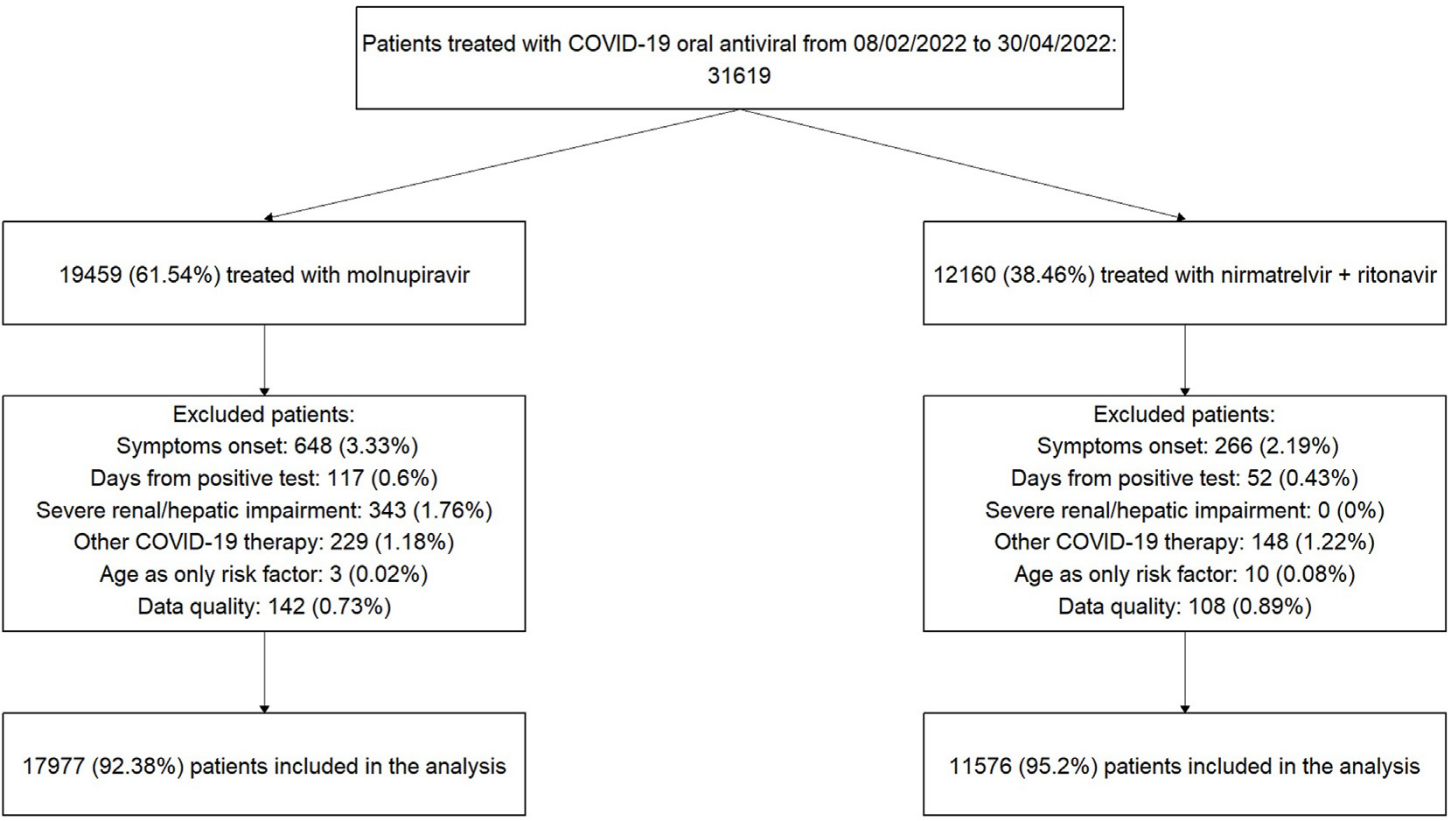
Study profile.

The flow chart of the patients included in the study is depicted in Fig. 1. In the considered timeframe, 31,619 patients were registered in the wMRs (61.5% treated with molnupiravir and 38.5% treated with nirmatrelvir plus ritonavir). After exclusion of patients who did not meet the inclusion or the quality control criteria, 17,977 patients were prescribed molnupiravir and 11,576 patients were prescribed nirmatrelvir plus ritonavir, all included in the analysis.

Patient characteristics are described in Table 1. In the overall population, almost an equal number of male (50.1%) and female (49.9%) patients were included. Patients treated with molnupiravir were older (median age: 74 years; 47.1% > 75 years old) than those treated with nirmatrelvir plus ritonavir (median age: 66.3 years; 29.1% > 75 years old). Median time from symptom onset was 3 days in both treatment groups. Most patients received SARS-CoV-2 vaccination (91.8%) with a full vaccine course including the booster dose (86.7%). Median number of co-morbidities was one in both treatment groups (1st–3rd quartiles were 1–2 and 1–1 in the molnupiravir and nirmatrelvir plus ritonavir cohort, respectively). Specifically, mild impairment of renal function, chronic kidney disease, uncontrolled diabetes, cardio-cerebrovascular diseases and severe pulmonary diseases were more frequent in patients who received molnupiravir, while primary or secondary immunodeficiencies and (haemato)-oncological diseases were more frequent in those who received nirmatrelvir plus ritonavir. Patients had mostly mild COVID-19 in both groups (85.5% overall). Distribution of signs and symptoms is reported in Supplementary Table S3.

**Table 1:** Baseline characteristics of treated patients.

Characteristics	Molnupiravir	Nirmatrelvir plus ritonavir	All
Sex Female	8714 (48.47%)	6027 (52.06%)	14,741 (49.88%)
Sex Male	9263 (51.53%)	5549 (47.94%)	14,812 (50.12%)
Age median (1st–3rd q)	74.06 (62.77–82.70)	66.31 (53.92–76.91)	71.29 (58.95–81.07)
Age <55	2501 (13.91%)	3114 (26.90%)	5615 (19.00%)
Age 55–75	7015 (39.02%)	5094 (44.00%)	12,109 (40.97%)
Age >75	8461 (47.07%)	3368 (29.09%)	11,829 (40.03%)
Days from symptoms onset median (1st–3rd q)	3 (2–4)	3 (2–3)	3 (2–4)
Days from positive test result median (1st–3rd q)	2 (1–3)	1 (1–2)	2 (1–2)
Vaccination–None or not completed	2288 (12.73%)	1648 (14.24%)	3936 (13.32%)
Fully vaccinated	15,689 (87.27%)	9928 (85.76%)	25,617 (86.68%)
Other	139 (0.77%)	81 (0.70%)	220 (0.74%)
ChAdOx1	279 (1.55%)	217 (1.87%)	496 (1.68%)
BNT162b2	13,372 (74.38%)	8192 (70.77%)	21,564 (72.97%)
JNJ-78436735	21 (0.12%)	27 (0.23%)	48 (0.16%)
mRNA-1273	1878 (10.45%)	1411 (12.19%)	3289 (11.13%)
Disease severity Mild	15,392 (85.62%)	9890 (85.44%)	25,282 (85.55%)
Disease severity Moderate	2585 (14.38%)	1686 (14.56%)	4271 (14.45%)
Saturation median (1st–3rd q)	97 (95–98)	97 (96–98)	97 (96–98)
Comorbidities median (1st–3rd q)	1 (1–2)	1 (1–1)	1 (1–2)
Non-Covid 19 related Oxygen-therapy	679 (3.78%)	299 (2.58%)	978 (3.31%)
Renal function mild impairment	898 (5.00%)	223 (1.93%)	1121 (3.79%)
Renal function moderate impairment	951 (5.29%)	675 (5.83%)	1626 (5.50%)
Hepatic function mild impairment	134 (0.75%)	74 (0.64%)	208 (0.70%)
Hepatic function moderate impairment	32 (0.18%)	21 (0.18%)	53 (0.18%)
BMI 30 kg/m^2^ or more	3603 (20.04%)	2756 (23.81%)	6359 (21.52%)
Chronic kidney disease	1520 (8.46%)	464 (4.01%)	1984 (6.71%)
Uncontrolled diabetes	2678 (14.90%)	1211 (10.46%)	3889 (13.16%)
Primary or secondary immunodeficiency	2706 (15.05%)	2579 (22.28%)	5285 (17.88%)
Cardio-cerebrovascular disease	9373 (52.14%)	3618 (31.25%)	12,991 (43.96%)
Severe pulmonary disease	3505 (19.50%)	2038 (17.61%)	5543 (18.76%)
Oncological disease	2525 (14.05%)	2328 (20.11%)	4853 (16.42%)

The crude incidence rate of all-cause mortality at day 28 was 40.23 per 100,000 person-days among the overall population. A higher crude incidence rate of all-cause mortality was found among molnupiravir users (51.83 per 100,000 person-days), compared to nirmatrervir/ritonavir users (22.29 per 100,000 person-days).

Univariable, age-adjusted and multivariable mixed effect Cox proportional hazard models were adopted to address whether the associations of baseline characteristics with mortality regardless type of treatment provided confirmation of the consistency of our cohort data with existing evidence available from the current literature. The results of this analysis are reported in Supplementary Table S4.

Propensity scores estimated from the gbm algorithm were used as weights to account for selection assignment differences between treatments. Their distributions are reported in Supplementary Figure S2 showing moderate overlap between the two groups. Still good covariate balance was obtained in term of absolute mean differences as illustrated in Supplementary Figures S4 and S5 and Supplementary Table S1. Before balancing, four covariates (age, number of co-morbidities, cardio- and cerebrovascular diseases and oxygen saturation) showed an absolute standardized mean difference higher than 0.1 which is generally considered a sign of imbalance.^35^ Of the four, the age factor was particularly challenging in consideration of how strongly associated it was to the risk of death by day 28 (Supplementary Table S4). After weight-adjusting, no covariates showed an absolute standardized mean difference higher than 0.05 with age showing a difference of 0.03 and very similar density distributions (Supplementary Figure S4).

**Fig. 2:**
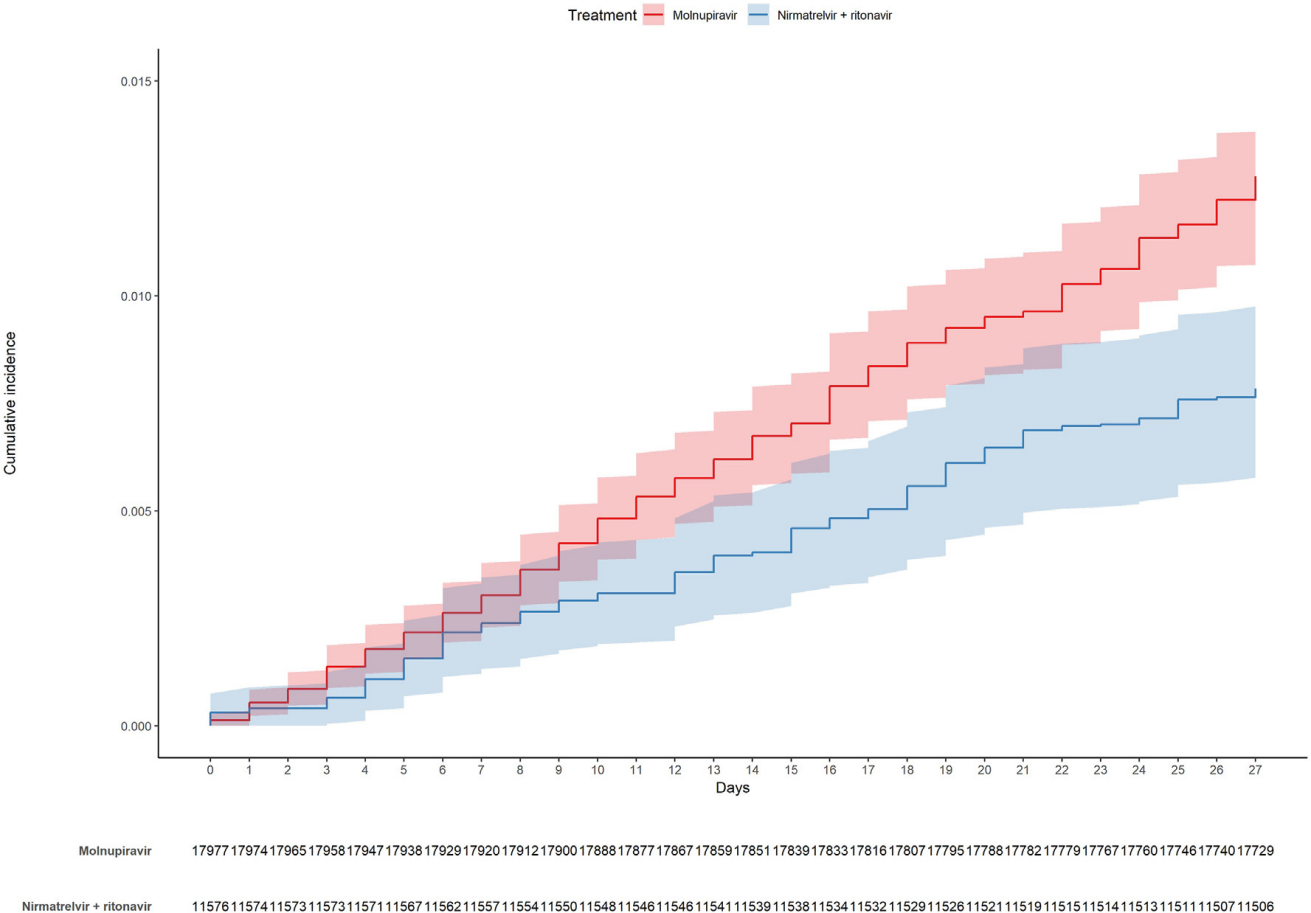
Cumulative incidence plot (weight-adjusted).

**Fig. 3:**
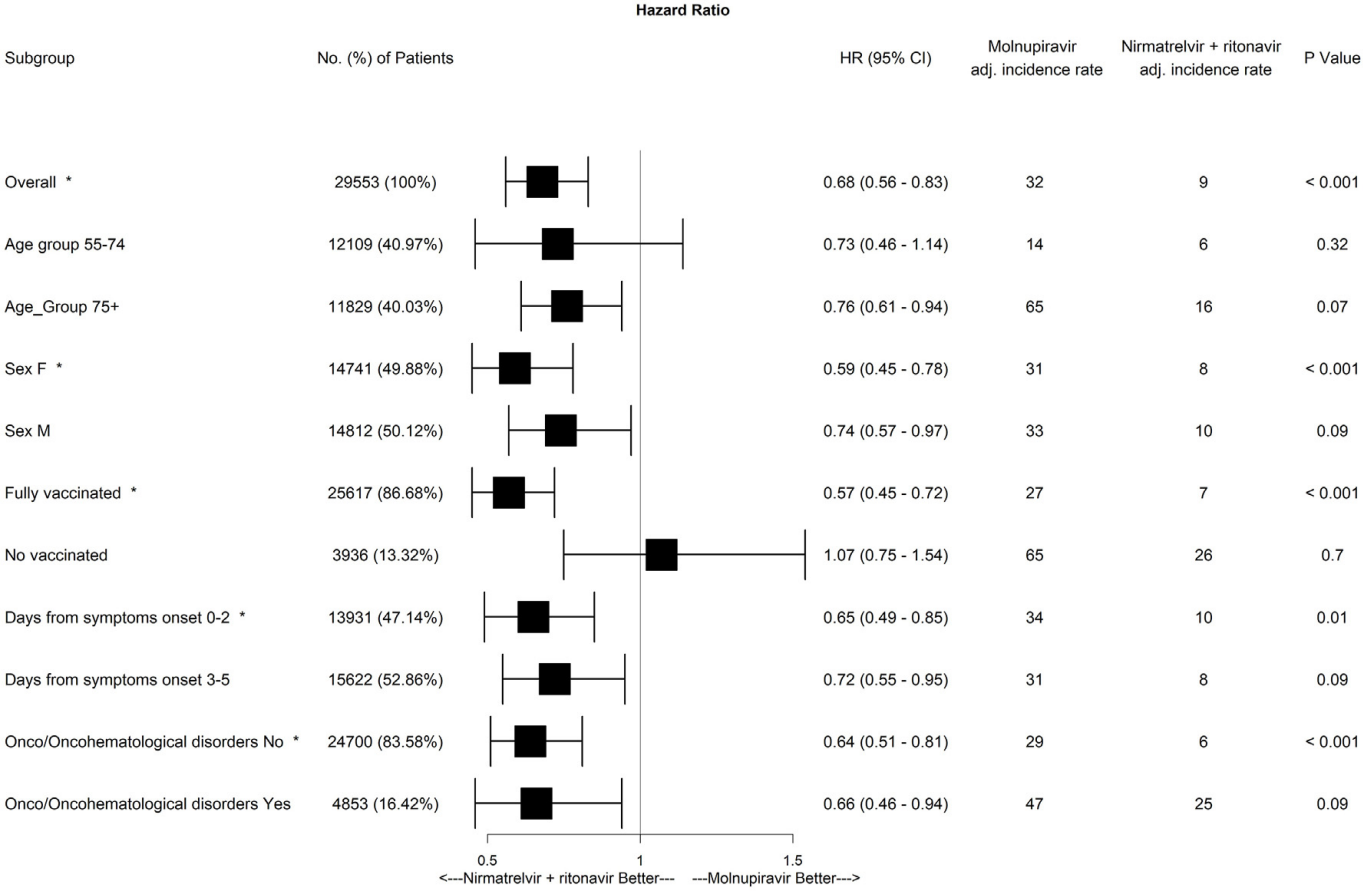
Hazard ratios for death after 28 days since drug administration (nirmatrelvir plus ritonavir compared to molnupiravir) in the overall population of patients and in subgroups of selected variables.

Subsequently, we compared the weight-adjusted cumulative incidences in the molnupiravir and nirmatrelvir plus ritonavir groups (Fig. 2) using the Aalen estimator. By day 28, adjusted cumulative incidence rates were 1.23% (95% CI 1.07%–1.38%) and 0.78% (95% CI 0.58%–0.98%) for molnupiravir and nirmatrelvir plus ritonavir, respectively (adjusted log rank p = 0.0002). Moreover, to account for possible differences among patients treated in different NHS centers, we also performed a weight-adjusted mixed-effect Cox model including Italian regions and NHS centers as random effects and treatment as the only covariate. Nirmatrelvir plus ritonavir was associated to a reduction in the risk of death by day 28 compared to molnupiravir (HR 0.68 [95% CI 0.56–0.83], see Fig. 3). To assess treatment effect on specific sets of patients, we also performed separated analysis on the following subgroups of patients: i) in the range of 55–74 years; ii) aged 75 years or more; iii) male; iv) female; v) fully vaccinated; vi) non-vaccinated or partially vaccinated; vii) treated up to 2 days since symptoms onset; viii) treated from 3 to 5 days since symptoms onset; ix) (haemato)-oncological diseases; x) no (haemato)-oncological diseases.

After adjusting for multiple comparisons, a significant reduction in the risk of death, calculated by Cox models and associated with the use of nirmatrelvir plus ritonavir vs molnupiravir was identified in female patients (p < 0.001), fully vaccinated patients (p < 0.001), patients treated up to 2 days since symptom onset (p = 0.01) and patients with no (haemato)-oncological diseases (p < 0.001) (Fig. 3). In all cases, patient variability among regions and NHS centers was taken into account including random effects in the models. Moreover, covariate balance in all subgroups was analyzed by visual inspection of the Connect-S plot.^36^ No imbalance was identified, supporting the theory that tree-based methods might produce appropriate covariate balance also across subgroups without pre-specifying possible interactions.^37^^,^^38^

Regarding tolerability, among 11,432 patients treated with molnupiravir for whom prescribing physicians filled in the follow-up forms completing data on adverse events (Supplementary Figure S8), 471 (4.1%) reported at least 1 adverse drug reaction, while 93 (0.8%) reported at least two. The corresponding rates for the 6602 patients treated with nirmatrelvir plus ritonavir with complete follow-up information (Supplementary Figure S8) were 11.4% (755/6602) and 3.0% (199/6602), respectively. Among 581 total events recorded in patients treated with molnupiravir, the three most frequent reactions were diarrhea (26.7%), nausea (22.2%), and dizziness (9.1%). By contrast, among 1016 events in patients treated with nirmatrelvir plus ritonavir, the three most frequent were dysgeusia (37.0%), diarrhea (16.7%) and nausea (14%).

## Discussion

Early initiation of nirmatrelvir plus ritonavir was associated to a reduced risk of all-cause mortality in comparison to molnupiravir in this nationwide cohort of patients with COVID-19 during a pandemic period dominated by the SARS-CoV-2 Omicron variants or subvariants.

Early RCTs demonstrated a significant reduction in COVID-19-related hospitalization or death with nirmatrelvir plus ritonavir or molnupiravir compared to placebo,^5^^,^^6^ leading to emergency authorisation of both drugs. However, RCTs were conducted in largely unvaccinated patients, in non-immunocompromised patients, and well before the emergence of the Omicron variants or subvariants. Moreover, since these trials were designed to compare study drugs with placebo, they did not provide direct comparisons of nirmatrelvir plus ritonavir with molnupiravir. Lastly, RCTs were conducted in quite homogeneous and selected populations. For the forementioned reasons, more real-life data are needed to be investigated in older, racially, and ethnically diverse populations with a high prevalence of underlying conditions to support and compare treatment effectiveness of the two available drug options for oral treatment of SARS-CoV-2 infected outpatients or inpatients for reasons unrelated to COVID-19.

To the best of our knowledge, the present study provides the first adequately powered results showing a statistically significant clinical benefit on short term mortality for COVID-19 patients treated with nirmatrelvir plus ritonavir over molnupiravir in the Omicron era. Indeed, three studies with adequate statistical power investigated the relative effectiveness of nirmatrelvir plus ritonavir compared to molnupiravir after adjustments for potential confounders.^23^, ^24^, ^25^ However, none of them produced solid and statistically significant results supporting the real benefit of one drug over the other in terms of patient survival both in the outpatient and in the inpatient setting.^23^, ^24^, ^25^

In fact, Bajema KL et al.^23^ used a target trial emulation approach to the analysis of observational data to compare nirmatrelvir plus ritonavir versus molnupiravir. Compared with molnupiravir-treated patients, those who were treated with nirmatrelvir plus ritonavir showed a reduction in absolute risk of death (−7.89 events per 1000 persons, 95% CI −15.00 to −0.61), while the relative risk of death between the two groups did not reach statistical significance (RR 0.14, 95% CI 0.02–1.16). Paraskevis D. et al.^24^ found a lower relative risk of death in patients on nirmatrelvir plus ritonavir compared to those on molnupiravir, although it appeared to be marginally not significant (OR 0.69, 95% CI 0.46–1.06; p = 0.09). In this study, the effect size was smaller but the relative risk was higher compared to Bajema et al.^23^ Moreover, since patients were selected in two different periods (those on molnupiravir between February 2, 2022 and March, 5, 2022, while those on nirmatrelvir plus ritonavir between March 26, 2022 and July 20, 2022), some unmeasured confounder biases such as different circulation of SARS-CoV-2 variants or subvariants may have influenced the results. By contrast, our study was conducted within less than three months, meaning that, even though a viral sequence analysis was not performed at individual level, in such a restricted period of time the SARS-CoV-2 sequences in Italy were well characterized at population levels,^26^ and quite uniformly distributed among BA.1, BA.1.1 and BA.2. Lastly, Wai et al.^25^ analysed real-world data from the Hospital Authority in Hong Kong to evaluate all-cause mortality and economic implications of prescribing oral antivirals. Among high-risk patients with mild to moderate COVID-19 in the inpatient cohort, the use of both nirmatrelvir plus ritonavir (HR 0.10 [95% CI 0.05–0.21], p < 0.0001) and molnupiravir (HR 0.31 [95% CI 0.24–0.40], p < 0.0001) was significantly associated with a reduced all-cause mortality without any statistically significant differences between the two groups. Unfortunately, in the outpatient cohort, a survival analysis was not performed due to the small number of clinical events. Conclusively, our results add relevant clinical information to the literature on real-world effectiveness of oral antiviral agents against SARS-CoV-2. Indeed, the present study is the first among the adequately powered studies to demonstrate an additional clinical benefit of nirmatrelvir plus ritonavir on patient survival in a population-based, nationwide cohort of mainly vaccinated patients during Omicron variant and subvariant circulation.

Analysis of the association between selected baseline characteristics and death was not the primary goal of this study. Moreover, we are aware that studying the association between factors and outcome should follow a specific methodology to avoid misinterpretation of the findings. Notwithstanding these limitations, univariable mixed effect Cox proportional hazard models for the association between selected baseline characteristics and mortality by day 28 were conducted in the overall population without considering the received treatment as well as bivariate analyses were conducted adjusting each variable for age (Supplementary Table S4–Bivariable and Multivariable). The results of these analyses were confirmed by a multivariable model, supporting the consistency of our cohort data with existing evidence available from the current literature. In fact, we found an increased risk of death in older patients and in those with co-morbidities such as chronic kidney disease, cardio and cerebrovascular and (haemato)-oncological diseases as it was previously demonstrated.^2^^,^^3^^,^^39^^,^^40^ Quite unexpectedly, BMI ≥30 kg/m2 appeared to be associated with a lower risk of death. Conflicting data exists concerning obesity, in particular intermediate levels of BMI, and risk of adverse outcomes in COVID-19 patients, whereby the underlying reasons are still a growing matter of debate.^41^^,^^42^ Lastly, associations between vaccination, timing of antiviral therapy, severity of the disease and the risk of death are not unexpected and confirm the current evidence available from literature, in particular with regard to the importance of vaccination.^43^

Of note, reduced risk of mortality associated with the use of nirmatrelvir plus ritonavir compared to molnupiravir was consistently observed in the overall population and in most of the patient subgroups, reinforcing the additional benefit of early use of nirmatrelvir plus ritonavir over molnupiravir in reducing all-cause mortality. However, statistical significance using a stringent method (Holm-Bonferroni correction) was only met in female patients, those who received a full vaccination course, who started antiviral treatment ≤2 days from symptom onset, in addition to those who did not suffer from (haemato)-oncological diseases. This suggests that in patients with greater risk of clinical progression, such as those not fully vaccinated or affected by (haemato)-oncological diseases, the decreased risk of death associated to using nirmatrelvir plus ritonavir rather than molnupiravir was not observed though HRs were <1 in all subgroups except from in the subgroup of patients who did not receive a full vaccination course before Holm-Bonferroni correction. We cannot however exclude that other factors influencing the risk of death in these categories were not available for adjustment and were not related to the selected treatment, as well as that a lower numerosity in both the registered events and the subgroup sample size have had some sort of impact on the outcome observed.

Although the analysis was based on data reported in end-of-treatment forms which were unavailable for a significant number of patients at freezing of the database and underreporting cannot be excluded, tolerability was assessed in 18,042 patients in this study. In this non-representative sub-cohort, at least one side effect was reported in 6.8% patients, a closer value to those available from the clinical trials rather than to others observed in similar real-world studies. As already shown,^44^ the proportion of patients who experienced any side effects was higher in patients belonging to the nirmatrelvir plus ritonavir group (11.4%) compared to the molnupiravir one (4.1%). Therefore, albeit incomplete, data seems to confirm the hypothesis of a better tolerability of molnupiravir compared to nirmatrelvir plus ritonavir, one of the reasons underlying the decision of the National Institutes of Health and Infectious Diseases Society of America guidelines,^45^^,^^46^ to consider molnupiravir as an alternative COVID-19 therapy in high-risk, non-pregnant adults without access to nirmatrelvir plus ritonavir or remdesivir or with contraindications to their use (*i.e.*, unmanageable drug–drug interactions or severe end-organ dysfunction).

The present study has some strengths and limitations. One important strength is related to the representativeness of the cohort and completeness of the study outcomes. In fact, enrollment of the patients took place throughout the entire Italian territory and all patients were captured because inclusion in the registry was compulsory to get the authorization to prescribe the study drugs. Moreover, all inpatient and outpatient deaths were recorded through a cross-check with the National Death Registry provided by the Ministry of the Interior. As a result, all patients were included in the database nonetheless those whom the clinicians were unaware of their conditions since they never returned for follow-up. Study limitations are mainly related to its observational nature. First, treatment groups were unbalanced, therefore a gbm algorithm was applied and mixed effect models were performed to account for the underlying variations between Italian regions and among the NHS centers. Second, only death was considered, while other studies considered death or hospital admission as a composite outcome. However, we decided to focus only on death to provide a stronger measure. Third, since there can be a correlation between the unhealthier baseline condition of patients receiving molnupiravir and the analysis of the mortality by all-cause, the comparative results between molnupiravir and nirmatrelvir plus ritonavir could have been changed if one had considered only the mortality associated to COVID-19. Unfortunately, it was not possible to differentiate COVID-19 from other diseases or complications as cause of death, which is intrinsically very difficult for such a complex disease such as COVID-19, especially in elderly patients affected by several comorbidities. Along the same line, being hospitalised was not an exclusion criterium provided that the reason for hospitalisation was unrelated to COVID-19 and information about hospitalisation and reasons for it were not recorded, while they could have been useful as a proxy of severe patient conditions. However, to reduce the possible bias, we limited the follow-up to 28 days to provide a more direct correlation of death with COVID-19 and treatments under study. Since extension to six-month follow-up would provide a total of 1255 events, accounting for a crude incidence rate of 23.81 events per 100,000 person-days (data not shown), it would be interesting to explore the possible predictors of this longer-term outcome. For this purpose, a further study will be conducted using a more in-depth assessment of the causes of death and a more robust methodology. Indeed, deaths occurring after more than one month from SARS-CoV-2 infection are more likely to be attributable to causes different from COVID-19 compared to deaths occurring earlier. Fourth, the analysis of toxicity/tolerability events were merely exploratory since they were recorded only in a subset of patients. Fifth, a control group of untreated patients with antivirals were not included, therefore we are not able to support the effectiveness of either molnupiravir or nirmatrelvir plus ritonavir to reduce mortality in our populations. Sixth, no data was available in the AIFA registry on time to SARS-CoV-2 viral clearance. The positive nasopharingeal swab result was considered as one of the baseline criteria to include patients but it was not requested either during follow-up or at day 28 final report.

At the completion of this study, the European Medicines Agency have recommended the refusal of the marketing authorization for molnupiravir^47^ and, following this negative opinion, the Scientific Technical Commission of AIFA decided to suspend the use of molnupiravir.^48^ An apparent reduced effectiveness of molnupiravir compared to nirmatrelvir plus ritonavir at least does not disprove the decision, however our exploratory analysis on follow-up reports seems to highlight a better profile of tolerability for molnupiravir compared to nirmatrelvir plus ritonavir, despite the older age registered in the molnupiravir group. Moreover, since our study lacks a comparable group of untreated patients, the results should not be interpreted as lack of effectiveness of molnupiravir treatment. In apparent contrast to the results of the PANORAMIC trial,^49^ which was not able to demonstrate any significant efficacy of molnupiravir, a positive effect of this drug was found on hospital admission or death,^19^ and on severe COVID-19 and COVID-19 related mortality particularly in specific subgroups.^22^ Therefore, although nirmatrelvir plus ritonavir is recommended as the preferred option compared to molnupiravir,^45^^,^^46^ more strategic studies should be conducted for better evidence-based guidelines.

In conclusion, in this prospective, nationwide cohort, of outpatients or inpatients hospitalised for reasons other than COVID-19, early initiation of nirmatrelvir plus ritonavir was associated with a significantly reduced risk of death compared to molnupiravir. Reduced risk of mortality was consistently observed in the overall population and in most patient subgroups, such as in female patients, fully vaccinated patients who received the booster dose, patients treated up to 2 days since symptom onset, and patients without (haemato)-oncological diseases, reinforcing generalizability of the results.

## Contributors

The study was designed by Carlo Torti, PPO, SV, PB, Carlo Tascini, GDP, PR, GP. Data analysis were done by PPO and verified by SC, AS, AV. Data were collected by Carlo Torti, PB, Carlo Tascini, GDP, DT, EN, ET, BC, AP, GBB, GP, MB, CB, AG, EME, MF, ML. Carlo Torti, PPO, PB, Carlo Tascini e GDP wrote the first draft of the manuscript which was revised by EN, PR and GP. All authors interpreted data, provided critical review and revision of the text, and approved the final version of the manuscript. PPO, SC, SV, PR and GP had access to the data.

## Data sharing statement

Data were obtained from an administrative database and sharing is not applicable due to legal issues.

## Declaration of interests

**PPO**, **SC**, **VS**, **PR**, **Giorgio Palù**, **Carlo Torti**, **AS**, **AV, DT, ET, AP, CB, AG, EME, MF, GBB and ML** report no competing interests regarding this article.

**PB** reports research grants and/or personal fees for advisor/consultant and/or speaker/chairman from Viiv, Gilead, Jannsen, Merck and Pfizer.

**CT** reports research grants and/or personal fees for advisor/consultant and/or speaker/chairman from Gilead, Merck, Pfizer, Menarini, GSK, Sanofi, Angelini, thermofischer, Biotest and Diasorin.

**EN** reports research grants and/or personal fees for advisor/consultant and/or speaker/chairman from Gilead, Eli Lilly, Roche, SOBI.

**BC** reports research grants and/or personal fees for advisor/consultant and/or speaker/chairman from Angelini, Menarini.

**Giustino Parruti** reports research grants and/or personal fees for advisor/consultant and/or speaker/chairman from Gilead, Merck, AlphaSigma, Angelini, Pfizer, Lusofarmaco, GSK, Janssen.

**MB** reports research grants and/or personal fees for advisor/consultant and/or speaker/chairman from Angelini, BioMérieux, Cidara, Menarini, MSD, Pfizer and Shionogi.

**GDP** reports research grants and/or personal fees for advisor/consultant and/or speaker/chairman from GS, MSD, ViiV, Abbvie, Janssen, GSK, AZ, Pfizer, Roche.
