# Supplementary material for: Real-life comparison of mortality in patients with SARS-CoV-2 infection at risk for clinical progression treated with molnupiravir or nirmatrelvir plus ritonavir during the Omicron era in Italy: a nationwide, cohort study
Source: Lancet Reg Health Eur. 2023 Jul 14;31:100684. doi: 10.1016/j.lanepe.2023.100684 (PMC10398591; doi:10.1016/j.lanepe.2023.100684)

**Supplementary Materials (in order of appearance)**

**Summary (in order of appearance)**

1. **Supplementary Figure S1:** Convergence of the gradient boosted model
2. **Supplementary Figure S2:** Distribution of propensity scores for molnupiravir and nirmatrelvir plus ritonavir
3. **Supplementary Figure S3:** Distribution of weights
4. **Supplementary Figure S4:** Distributions and proportions of demographic variables and clinical variables
5. **Supplementary Figure S5:** Standardized mean differences before and after adjustment (overall population)
6. **Supplementary Table S1:** Standardized mean differences before and after adjustment (overall population) – Complete table
7. **Supplementary Figure S6:** Standardized mean differences before and after adjustment (subgroups)
8. **Supplementary Figure S7:** Beta(t) over time for variable (Haemato)-Oncological disease in the univariable, Age-adjusted and multivariable Cox proportional hazard models
9. **Supplementary Table S2:** Independence test between Schoenfeld residuals and time in univariable, age-adjusted and multivariable Cox proportional hazard models
10. **Supplementary Table S3:** Distribution of signs and symptoms
11. **Supplementary Table S4:** Univariable, Age adjusted and multivariable mixed-effect Cox proportional hazard models for baseline Characteristics and mortality by day 28. The multivariable model included all baseline characteristics reported in Supplementary Table
12. **Supplementary Figure S8:** Flowchart and exclusion criteria of patients for whom end-of-follow-up reports were filled in and tolerability profiles were available

**Supplementary Figure S1 –** Convergence of the gradient boosted model

The figure shows the average standardized effect size and the maximum Kolmogorov-Smirnov (KS) statistic as a function of the number of trees. Minimal differences between molnupiravir and nirmatrelvir+ritonariv is achieved with about 4872 trees in the case of standardized effect size and 4574 trees for max. KS statistic. Since the minima achieved are similar, results should be robust to the stopping rule adopted for the analysis (average standardized effect size).

**Supplementary Figure S2 –** Distribution of propensity scores for molnupiravir and nirmatrelvir + ritonavir

The figure shows the distributions of propensity scores for the molnupiravir cohort (treatment 1) and for the nirmatrelvir + ritonavir cohort (treatment 2).

**Supplementary Figure S3 -** Distribution of weights

Histogram of the weight distribution of the overall cohort.

**Supplementary Figure S4 -** Distributions and proportions of demographic variables and clinical variables before and after adjustment of the main clinical and demographic variables included in the propensity score estimation. Molnupiravir cohort is red-colored, nirmatrelvir+ritonavir cohort is blue-colored. Clinical variables were selected considering the hazard ratios shown in figure 2. Similar consideration was adopted for the Age (years) variable while gender was included for its well-known role as confounder. Adjusted distributions and proportions were reported for both stopping rule tested (es.mean and ks.max).


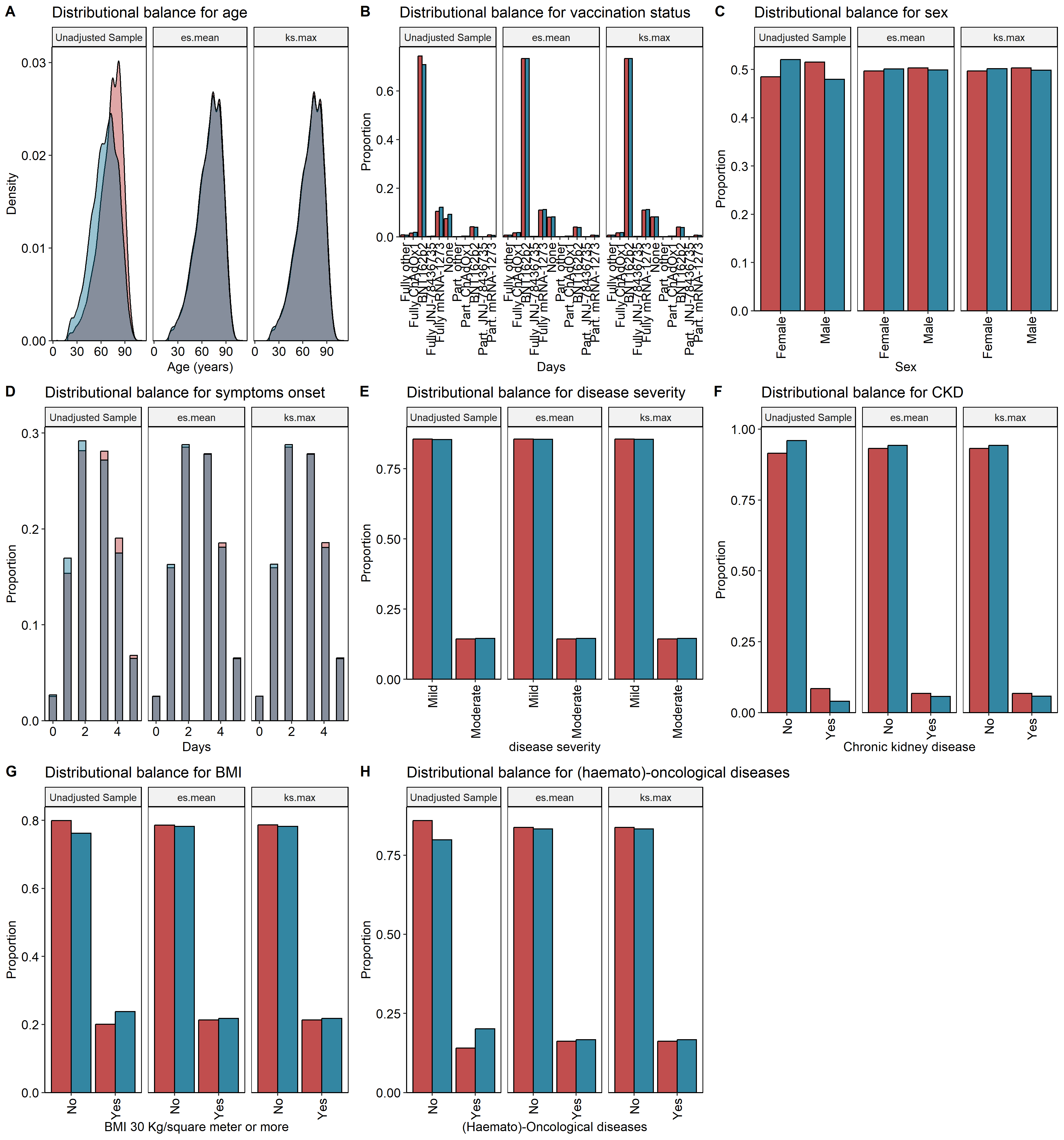


**Supplementary Figure S5 -** Standardized mean differences before and after adjustment (overall population)


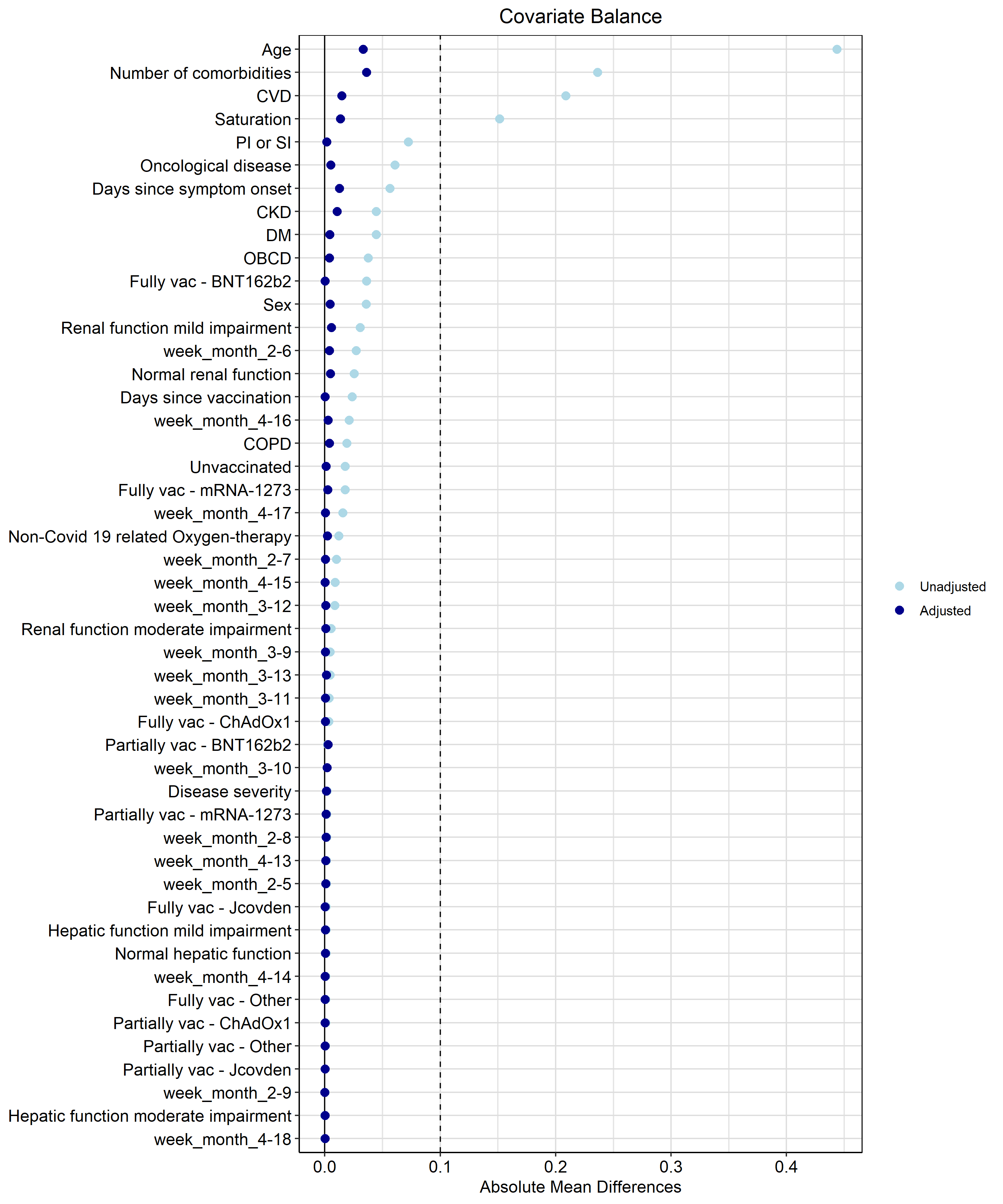


Absolute standardized mean differences before and after adjustment. The dotted line represents the 0.1 threshold which is generally considered a sign of imbalance.

|  |  |  |  |  |
| --- | --- | --- | --- | --- |

**Supplementary Table S1** - Standardized mean differences before and after adjustment (overall population)

See Excel table Sup Tab 2.

**Supplementary table S2 -** P-values of the independence test between Schoenfeld residuals and time – Multivariable Cox proportional hazard model.

|  | **Univariable** | **Age-adjusted** | | **Multivariable** |
| --- | --- | --- | --- | --- |
| Sex | 0.07 | 0.07 | 0.07 | |
| Age group | 0.35 |  | 0.36 | |
| Days since symptoms onset | 0.08 | 0.08 | 0.08 | |
| Vaccination status | 0.10 | 0.11 | 0.11 | |
| Disease severity | 0.46 | 0.46 | 0.46 | |
| CKD | 0.27 | 0.27 | 0.27 | |
| SPD | 0.97 | 0.97 | 0.97 | |
| CVD | 0.09 | 0.09 | 0.09 | |
| DM | 0.25 | 0.24 | 0.24 | |
| BMI | 0.61 | 0.61 | 0.61 | |
| (Haemato)-Oncological disease | 0.02 | 0.02 | 0.02 | |
| Primary or acquired immunodeficiency | 0.08 | 0.07 | 0.07 | |
| Number of comorbidities | 0.64 | 0.65 | 0.64 | |
| Global |  |  | 0.11 | |

**Supplementary Table S3 –** Distribution of signs and symptoms

| **Symptoms** | **Molnupiravir** | **Nirmatrelvir + ritonavir** | **All** |  |
| --- | --- | --- | --- | --- |
| **Asthenia** | 6135 (34.13%) | 3654 (31.57%) | 9789 (33.12%) |  |
|  |  |  |  |  |
| **Myalgias** | 4494 (25.00%) | 2760 (23.84%) | 7254 (24.55%) |  |
|  |  |  |  |  |
| **Nasal congestion** | 4822 (26.82%) | 3006 (25.97%) | 7828 (26.49%) |  |
|  |  |  |  |  |
| **Fever** | 8443 (46.97%) | 6015 (51.96%) | 14458 (48.92%) |  |
|  |  |  |  |  |
| **Cough** | 10597 (58.95%) | 6858 (59.24%) | 17455 (59.06%) |  |
|  |  |  |  |  |
| **Headache** | 3107 (17.28%) | 2102 (18.16%) | 5209 (17.63%) |  |
|  |  |  |  |  |
| **Dyspnea** | 1120 (6.23%) | 610 (5.27%) | 1730 (5.85%) |  |
|  |  |  |  |  |
| **Gastrointestinal symptoms** | 686 (3.82%) | 450 (3.89%) | 1136 (3.84%) |  |
|  |  |  |  |  |
| **Anosmia** | 271 (1.51%) | 236 (2.04%) | 507 (1.72%) |  |
|  |  |  |  |  |
| **Pharyngodynia** | 5436 (30.24%) | 3719 (32.13%) | 9155 (30.98%) |  |
|  |  |  |  |  |
| **Shivers** | 892 (4.96%) | 435 (3.76%) | 1327 (4.49%) |  |

**Supplementary Table S4:** Univariable, Age-adjusted and multivariable mixed-effect Cox proportional hazard models for baseline Characteristics and mortality by day 28. The multivariable model included all baseline characteristics reported in Supplementary Table 3.

|  | **Univariable** | | | **Age-adjusted** | | | **Multivariable: all variables** | | |
| --- | --- | --- | --- | --- | --- | --- | --- | --- | --- |
|  | **HRs** | **LCL** | **UCL** | **HRs** | **LCL** | **UCL** | **HRs** | **LCL** | **UCL** |
| Sex: M vs F | 1.10 | 0.88 | 1.37 | 1.10 | 0.89 | 1.38 | 1.14 | 0.91 | 1.42 |
| Age group: 55-74 vs 54- | 5.06 | 2.16 | 11.88 |  |  |  | 5.29 | 2.25 | 12.47 |
| Age_Group: 75+ vs 54- | 20.87 | 9.13 | 47.69 |  |  |  | 21.47 | 9.28 | 49.66 |
| Days since symptoms onset | 0.90 | 0.82 | 0.99 | 0.91 | 0.83 | 1.00 | 0.91 | 0.83 | 1.00 |
| Vaccination: None or some vs Fully | 2.56 | 2.00 | 3.27 | 2.90 | 2.27 | 3.71 | 3.02 | 2.36 | 3.88 |
| Disease severity: Moderate vs Mild | 1.44 | 1.07 | 1.94 | 1.43 | 1.07 | 1.93 | 1.40 | 1.04 | 1.88 |
| Chronic kidney disease | 2.23 | 1.62 | 3.09 | 1.52 | 1.10 | 2.11 | 1.81 | 1.24 | 2.66 |
| Severe pulmonary diseases | 1.16 | 0.89 | 1.52 | 0.99 | 0.75 | 1.29 | 1.21 | 0.86 | 1.69 |
| Cardio and cerebrovascular disease | 1.42 | 1.14 | 1.78 | 0.84 | 0.67 | 1.06 | 1.09 | 0.80 | 1.50 |
| Uncontrolled diabetes | 0.82 | 0.57 | 1.16 | 0.72 | 0.51 | 1.03 | 0.88 | 0.58 | 1.32 |
| BMI 30 Kg/m^2 or more | 0.33 | 0.22 | 0.49 | 0.45 | 0.30 | 0.68 | 0.55 | 0.35 | 0.88 |
| Oncological disease | 2.08 | 1.62 | 2.66 | 2.40 | 1.87 | 3.09 | 2.53 | 1.81 | 3.54 |
| Primary or secondary immunodeficiency | 0.63 | 0.45 | 0.88 | 1.12 | 0.79 | 1.58 | 1.15 | 0.77 | 1.72 |
| Number of comorbidities: 3-4 vs 0-2 | 1.28 | 0.85 | 1.93 | 1.03 | 0.68 | 1.55 | 0.81 | 0.44 | 1.49 |
| Number of comorbidities: 5+ vs 0-2 | 3.04 | 0.41 | 22.73 | 2.24 | 0.30 | 16.76 | 2.00 | 0.22 | 18.21 |

Univariable mixed effect Cox proportional hazard models for the association between selected baseline characteristics and mortality by day 28 (only non-informative type 1 censoring) in the overall population without considering the received treatment. Increased risk was observed for age 55-74 years or >74 years vs <55 years, not having received a full vaccination course, moderate vs mild disease severity, chronic kidney disease, cardio and cerebrovascular disease and (haemato)-oncological diseases. By contrast, earlier initiation of oral antiviral therapy (for each day) from symptom onset and BMI ≥30 Kg/m2 appeared to be associated with a lower risk of death. After adjusting for age alone (bivariable model) or with all selected baseline characteristics (multivariable model), strength and direction of the observed associations were not affected except for Cardio and cerebrovascular disease.

**Supplementary Figure S6** - Standardized mean differences before and after adjustment (subgroups)

Connect-S plots **(Yang et al. Propensity score weighting for causal subgroup analysis; Statistics in Medicine 2021 DOI: 10.1002/sim.9029)** reporting absolute standardized differences before and after adjustment. Dots are colored from white to black, with darker shade implying more severe imbalance (see Austin and Stuart 2015). Column size refers to the number of patients in the given subgroup and VIF represents the variance inflation factor in the treatment effect estimates.

**Supplementary Figure S7** - Beta(t) over time for variable (Haemato)-Oncological disease


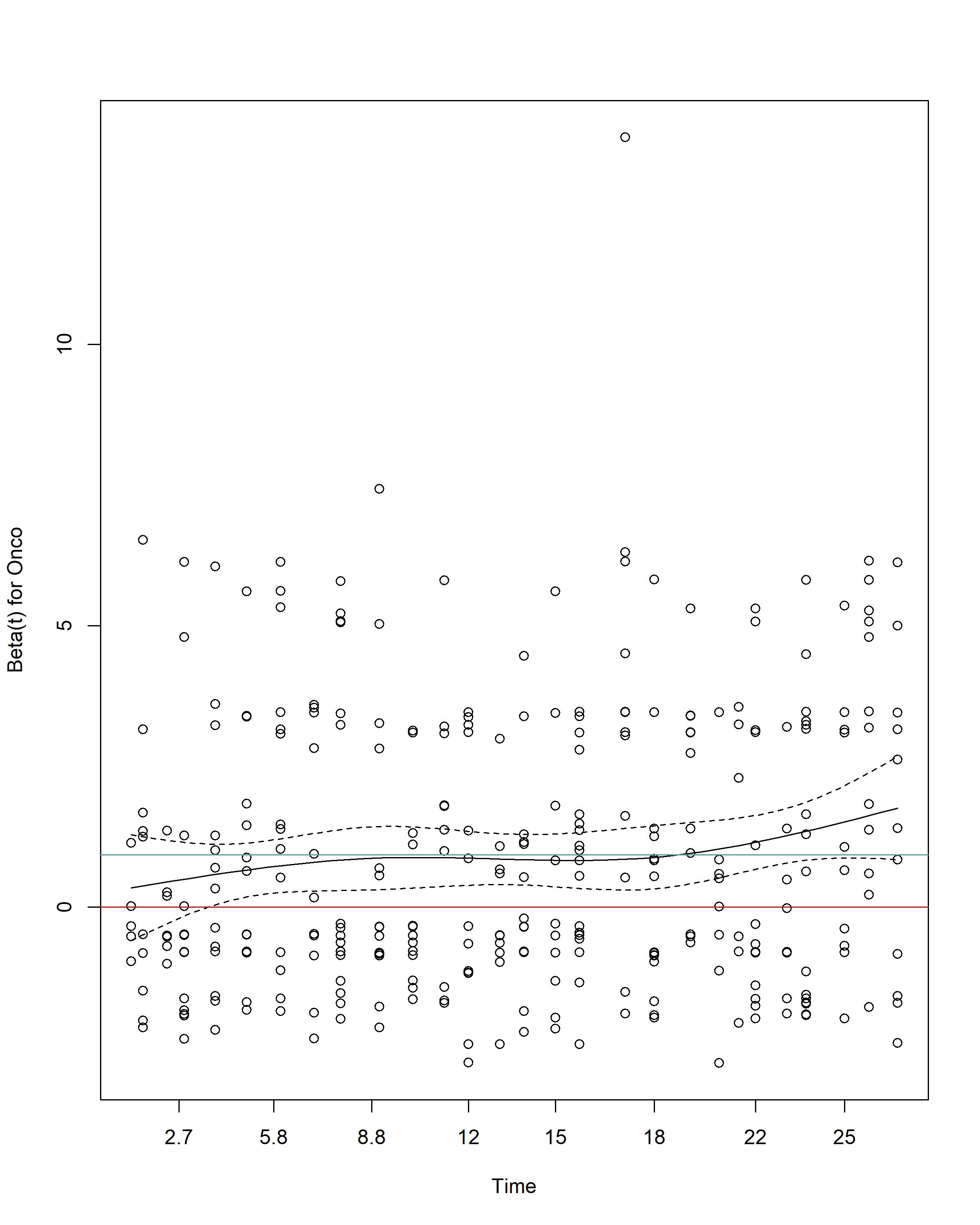


Visual inspection of beta as a function of time for variable (Haemato)-Oncological disease. Dots represents scaled Schoenfeld residuals, the solid line a smoothing spline curve fit to the plot and the black dotted lines a +/- 2-standard-error band. Zero coefficient is shown by the solid red-colored line, while the average coefficient over time is reported as a solid blue-colored line.

**Supplementary Figure S8** – Flowchart of patients with available end-of-treatment report. Non-compliant centers are defined as centers with a rate of uploaded end-of-treatment report equal to or higher than 75%. To avoid possible bias, derived from non at random missing data, only compliant centers were included in the tolerability analysis.


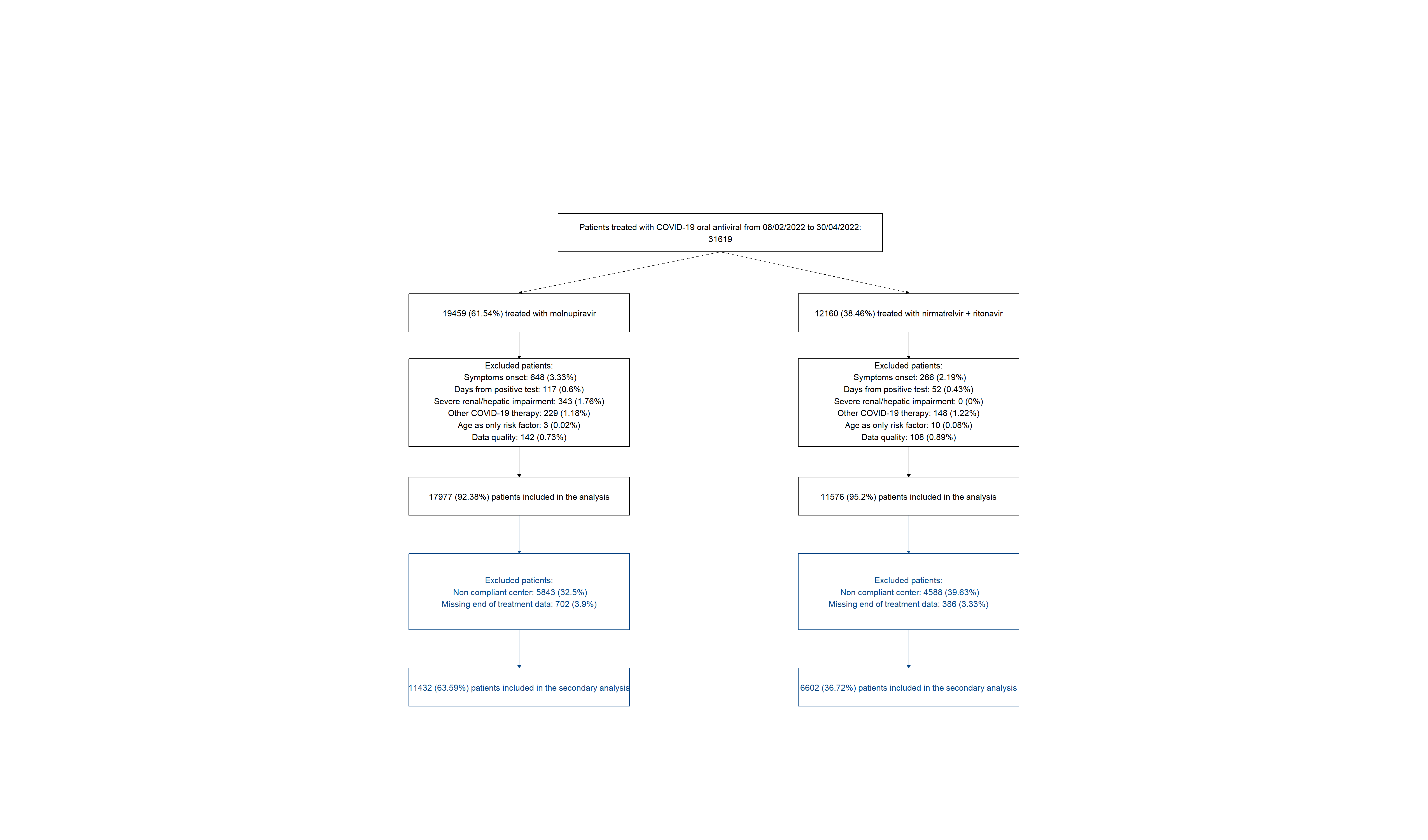

Supplement: Supplementary Figures and Tables [file mmc1.docx]
